# Editorial: New horizons in the management of advanced epithelial ovarian cancer

**DOI:** 10.3389/fmed.2026.2032855

**Published:** 2026-10-28

**Authors:** Orazio De Tommasi, Giulio Bonaldo, Roberto Tozzi

**Affiliations:** 1 Department of Women and Children's Health, Clinic of Gynecology and Obstetrics, University of Padua, Padua, Italy; 2 Department of Gynecological Oncology, Vall d’Hebron University Hospital, Universitat Autònoma de Barcelona, Barcelona, Spain

**Keywords:** advanced epithelial ovarian cancer, HIPEC, lymphadenectomy, minimally invasive surgery, molecular profiling, port-site metastasis, precision cytoreduction

Advanced epithelial ovarian cancer remains the most lethal gynecologic malignancy, with more than 324,000 new diagnoses and 206,000 deaths estimated worldwide in 2022 (1). Because symptoms are often nonspecific, most patients present with stage III-IV disease. Complete cytoreductive surgery and carboplatin-paclitaxel chemotherapy remain the therapeutic backbone, complemented by bevacizumab and poly(ADP-ribose) polymerase inhibitors according to clinical and molecular features. Nevertheless, approximately 70% of patients with advanced disease ultimately relapse (2).

For decades, the surgical paradigm was shaped by the observation that complete gross resection is among the strongest prognostic factors in advanced ovarian cancer (3). This association encouraged greater surgical effort and increasingly complex upper-abdominal and multivisceral procedures (4). However, technical feasibility does not necessarily establish an independent survival benefit, and much of the evidence supporting ultraradical surgery remains observational. The TRUST trial has partially reframed this debate. In quality-assured high-volume centers, primary cytoreductive surgery prolonged progression-free survival compared with neoadjuvant chemotherapy (NACT) followed by interval debulking surgery (IDS), but did not demonstrate statistically significant superiority for the primary overall-survival endpoint (5). This more selective paradigm may create space for minimally invasive surgery (MIS), particularly after an excellent response to NACT and in the presence of limited disease burden. In the ULTRA-LAP pilot study, laparoscopic visceral-peritoneal debulking was feasible in a selected subset of patients with stage III-IV ovarian cancer, with high complete-resection rates and favorable perioperative outcomes (6). The international LANCE trial is comparing MIS with laparotomy for IDS in patients with stage IIIC-IV high-grade epithelial ovarian cancer responding to three or four cycles of NACT. Its randomized lead-in phase reported conversion to laparotomy in 12.5% of evaluable patients assigned to MIS and complete gross resection in 88% of the MIS group and 83% of the laparotomy group (7). These findings established trial feasibility but cannot yet demonstrate oncologic non-inferiority. Until mature survival results are available, minimally invasive IDS should remain limited to carefully selected patients and expert surgeons, with prompt conversion whenever complete cytoreduction cannot be safely achieved.

The expansion of laparoscopy must nevertheless remain oncologically cautious. In this Research Topic, De Tommasi et al. combined a challenging case with the first systematic review examining port-site metastases (PSM) after diagnostic laparoscopy in advanced ovarian cancer. Reported incidence ranged from 1.18% to 46.7%. Advanced stage, extensive peritoneal disease, large-volume ascites, and treatment outside specialized centers emerged as recurrent risk factors. Routine port-site excision has not demonstrated a survival benefit and increases wound-related morbidity. Diagnostic laparoscopy should therefore remain embedded within standardized pathways emphasizing appropriate selection, meticulous technique, trocar-site management, and surveillance.

Precision surgery is equally relevant to lymph-node management. The LION trial demonstrated that systematic pelvic and para-aortic lymphadenectomy does not improve progression-free or overall survival in patients with complete intra-abdominal resection and clinically negative nodes, while increasing serious complications (8, 9). In this research topic, Xu et al. analyzed 654 patients and identified lymph-node metastases in 44.5%, with the left para-aortic region most frequently involved. Their Bayesian model described three principal conditional patterns linking iliac, common-iliac, para-aortic, obturator, inguinal, and presacral stations. Tumor size, histologic grade, and age were associated with metastatic extent. Although retrospective, this probabilistic map may support future risk-adapted nodal assessment.

Purwar et al. extend this concept from anatomy to biology. They propose that retroperitoneal nodes may function as pharmacologic and microenvironmental sanctuaries for resistant tumor clones. Their exploratory framework integrates homologous-recombination status with transcriptomic and proteomic resistance markers to identify a molecularly defined subgroup that might benefit from selective para-aortic clearance. The model is hypothesis-generating and should not alter current practice, but it illustrates how broad de-escalation could coexist with biology-driven escalation in prospective biomarker-stratified trials.

Hyperthermic intraperitoneal chemotherapy (HIPEC) represents another strategy integrating surgery with regional pharmacologic treatment. OVHIPEC-1 demonstrated a survival benefit when HIPEC was added to IDS in selected patients (10). In this Research Topic, Al Ameer et al. synthesized six randomized trials involving 773 patients and reported an overall-survival benefit in primary advanced disease, particularly in the IDS setting, without a significant pooled increase in postoperative complications; no clear survival benefit emerged in recurrent disease. HIPEC should therefore remain a selective modality delivered through standardized protocols in experienced centers.

Finally, precision oncology is becoming increasingly central to ovarian cancer management. Homologous recombination deficiency testing is already used to identify patients most likely to benefit from PARP-inhibitor maintenance, but current biomarkers do not fully capture prognosis, treatment sensitivity, or resistance evolution (11, 12). Complementary prognostic and predictive markers are therefore needed to refine risk stratification and tailor therapy. Zhang et al. identified TRIP13 as a progression-related biomarker associated with advanced-stage disease and shorter progression-free survival. Li et al. combined Mendelian randomization, bulk and single-cell transcriptomics, and experimental validation to identify NUP35, CASP3, BAG5, and DNAJB1 as integrated-stress-response-related prognostic genes in high-grade serous ovarian cancer. Although these signatures require prospective validation, they show how molecular models may refine therapeutic selection and potentially inform surgical decision-making.

In conclusion, primary debulking surgery and NACT followed by IDS should not be regarded as competing strategies, but as complementary options whose use should be individualized. The choice between PDS and IDS should integrate the anatomical distribution of disease, the probability of achieving complete gross resection, the anticipated complexity and morbidity of surgery, age, performance status, comorbidities, and patient preferences. Molecular characteristics, including BRCA mutation and homologous recombination deficiency status, may further contribute to this decision-making process as their relationships with tumor biology, chemosensitivity, resectability, and benefit from maintenance treatment become better defined. The future challenge is therefore to develop and prospectively validate integrated models capable of assigning each patient to the surgical timing and therapeutic sequence most likely to maximize survival while limiting unnecessary morbidity. We thank all authors and reviewers for their valuable contributions and hope that this Research Topic will stimulate further multidisciplinary research toward a more individualized management of advanced epithelial ovarian cancer.
